# Immunogenicity of Candidate MERS-CoV DNA Vaccines Based on the Spike Protein

**DOI:** 10.1038/srep44875

**Published:** 2017-03-23

**Authors:** Sawsan S. Al-amri, Ayman T. Abbas, Loai A. Siddiq, Abrar Alghamdi, Mohammad A. Sanki, Muhanna K. Al-Muhanna, Rowa Y. Alhabbab, Esam I. Azhar, Xuguang Li, Anwar M. Hashem

**Affiliations:** 1 Special Infectious Agents Unit, King Fahd Medical Research Center, King Abdulaziz University, Jeddah, Saudi Arabia; 2 Biotechnology Research Laboratories, Gastroenterology Surgery Center, Mansoura University, Mansoura, Egypt; 3 Hematology Laboratory, King Abdulaziz University Hospital, Jeddah, Saudi Arabia; 4 Materials Science Research Institute, National Nanotechnology Center, King Abdulaziz City for Science and Technology (KACST), Riyadh, Saudi Arabia; 5 Department of Medical Laboratory Technology, Faculty of Applied Medical Sciences, King Abdulaziz University, Jeddah, Saudi Arabia; 6 Center for Vaccine Evaluation; Biologics and Genetic Therapies Directorate; Health Canada; Ottawa, Ontario, Canada; 7 Department of Medical Microbiology and Parasitology, Faculty of Medicine, King Abdulaziz University, Jeddah, Saudi Arabia

## Abstract

MERS-coronavirus is a novel zoonotic pathogen which spread rapidly to >25 countries since 2012. Its apparent endemicity and the wide spread of its reservoir host (dromedary camels) in the Arabian Peninsula highlight the ongoing public health threat of this virus. Therefore, development of effective prophylactic vaccine needs to be urgently explored given that there are no approved prophylactics or therapeutics for humans or animals to date. Different vaccine candidates have been investigated but serious safety concerns remain over protein or full-length spike (S) protein-based vaccines. Here, we investigated the immunogenicity of naked DNA vaccines expressing different fragments of MERS-CoV S protein in mice. We found that plasmids expressing full-length (pS) or S1-subunit (pS1) could induce significant levels of S1-specific antibodies (Abs) but with distinct IgG isotype patterns. Specifically, pS1 immunization elicited a balanced Th1/Th2 response and generally higher levels of all IgG isotypes compared to pS vaccination. Interestingly, only mice immunized with pS1 demonstrated significant S1-specific cellular immune response. Importantly, both constructs induced cross-neutralizing Abs against multiple strains of human and camel origins. These results indicate that vaccines expressing S1-subunit of the MERS-CoV S protein could represent a potential vaccine candidate without the possible safety concerns associated with full-length protein-based vaccines.

Middle East respiratory syndrome coronavirus (MERS-CoV) is an emerging zoonotic pathogen recovered first from a fatal human case in Saudi Arabia in 2012^1^ and continued to infect almost 1800 people in over 25 countries. Saudi Arabia has reported the largest number of cases so far with cases continuing to increase. The virus causes severe respiratory infection associated with fever, cough, acute pneumonia, shortness of breath, systemic infection and occasional multi-organ failure in infected individuals leading to death in 35–40% of the cases^2^,^3^,^4^. Such a severe disease usually occurs in immunocompromised patients, individuals with comorbidities and the elderly^1^,^4^,^5^,^6^. Most of the reported MERS cases are linked to hospital outbreaks and family clusters due to close contact with infected patients^4^,^7^,^8^,^9^,^10^. However, accumulating epidemiological data show high prevalence of MERS-CoV in dromedary camels from several Arabian and African countries, suggesting that dromedaries might be the reservoir hosts of this virus^4^,^11^,^12^,^13^,^14^,^15^. The continued endemicity of MERS-CoV in the Arabian Peninsula and the associated high death rate clearly represent a public health concern with potential global spread as observed in the recent outbreak in South Korea^10^. This is further complicated by the lack of prophylactic or therapeutic measures, underscoring the importance of preparedness research against this potential pandemic virus.

Several supportive therapies and antivirals were proposed and examined for the treatment of MERS-CoV infections^16^,^17^,^18^,^19^,^20^. However, most of these strategies were based on the experience gained during the severe acute respiratory syndrome (SARS) outbreak or from MERS-CoV *in vitro* studies and require further preclinical and clinical evaluation. The ideal strategy to rapidly control existing and potential outbreaks of MERS-CoV is to generate a safe and effective vaccine at least to target high-risk groups or animal hosts. The ability of more than 60% of the infected patients to recover, clear the virus and develop immunity suggest that a vaccine based on the viral components such as the spike (S) glycoprotein could be a suitable vaccine candidate. This is further supported by the isolation of several human neutralizing antibodies (nAbs) against the MERS-CoV S protein and their ability to neutralize and block viral entry and/or cell-cell spread at very low concentrations, and sometimes to confer prophylactic and therapeutic protection in animal models^21^,^22^,^23^,^24^,^25^,^26^,^27^.

MERS-CoV S glycoprotein is composed of 2 subunits; the receptor binding domain (RBD) containing subunit (S1) and the fusion machinery subunit (S2)^28^. Several vaccines candidates based on full-length or truncated S protein were developed and investigated including DNA vaccines^29^,^30^, viral vectored vaccines^31^,^32^,^33^,^34^,^35^, nanoparticle-based vaccine^36^, whole inactivated MERS-CoV vaccine (WIV)^37^, as well as the S or RBD protein-based subunit vaccines^29^,^38^,^39^,^40^,^41^,^42^. While these experimental vaccines can induce protective response in animals, SARS-CoV vaccine development and a recent MERS-CoV report^37^ suggest that there might be serious safety concerns associated with the use of full length S protein as vaccine candidate including immunopathology and disease enhancement^43^,^44^,^45^,^46^,^47^,^48^. These concerns were proposed to be due to inductions of Th2- skewed immune response and/or anti-S non-neutralizing Abs.

DNA vaccines represent a promising vaccine development approach due to their easy production on a large scale in a timely manner and well-established procedures for quality control. In addition, DNA vaccines can elicit Th1-biased immune response in contrast to the protein-based subunit vaccines. However, all MERS-CoV DNA vaccines reported so far were aimed at expressing full-length protein, which could induce adverse reactions. In this study, we determined the immunogenicity and potential protective effects of MERS-CoV naked DNA vaccines expressing different length of S protein.

## Materials and Methods

### Cell line and MERS-CoV viruses

African Green monkey kidney-derived Vero E6 cells (ATCC #1568) were grown in Dulbecco’s modified Eagle’s medium (DMEM) supplemented with 10% fetal bovine serum (FBS), 1% penicillin/streptomycin, and 10 mM HEPES (pH 7.2) and maintained in a humidified 5% CO_2_ incubator at 37 °C. MERS-CoV strains used in this study included a human isolate (MERS-CoV/Hu/Taif/SA/2015) and two camel isolates (MERS-CoV/Camel/Taif/SA/31/2016 and MERS-CoV/Camel/Taif/SA/39/2016). MERS-CoV viruses were isolated, passaged and titrated by TCID_50_ in Vero E6 cells as previously described^49^. All tested isolates were at passage no. 2. All experiments involving live virus were conducted in our Biosafety level 3 facility following the recommended safety precautions and measures.

### DNA constructs

**Figure 1:**
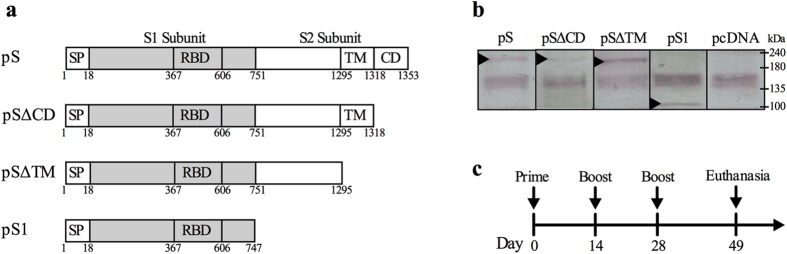
MERS-CoV Spike DNA vaccines. (**a**) Schematic representation of the generated DNA vaccine constructs. Four constructs were generated including one expressing full length S protein (pS) and three other constructs expressing truncated S protein with deleted cytoplasmic domain (pS∆CD), deleted transmembrane domain (pS∆TM) or deleted S2 subunit (pS1). Numbers indicate amino acids. SP: signal peptide; RBD: receptor-binding domain; TM: transmembrane domain; CD: cytoplasmic domain. (**b**) *In vitro* protein expression in cell culture. Vero E6 cells with 80–90% confluency were transfected with the DNA constructs; 48 h later, cell lysates were collected; protein expression was subsequently confirmed by western blot using anti-S1 polyclonal Abs. Arrows indicate band with expected molecular weight. (**c**) Time-line of immunization regimen.

Four DNA vaccine candidates were generated as shown in Fig. 1a. Full length MERS-CoV S gene from MERS-CoV-Jeddah-human-1 isolate (GenBank accession number: KF958702) was codon optimized for efficient mammalian expression and synthesized by Bio S & T (Montreal, Canada). The coding sequence was then subcloned into the mammalian expression vector pcDNA3.1 under the control of the cytomegalovirus immediate-early promoter generating pS construct. The second construct (pS1) expressing S1 domain (aa 1–747) was produced by cloning corresponding coding region by PCR using Phusion High-Fidelity PCR Kit (Life Technologies) from pS plasmid into pcDNA3.1 vector using the following forward 5′-GATCGCGGCCGCGCCACCATGATCCAC-3′ and reverse 5′-GATCGGTACCTTACAGAATGAAAAAGACGC-3′ primers. Similarly, pS∆TM expressing truncated S protein (aa 1–1295) without the transmembrane domain and pS∆CD expressing truncated S protein (aa 1–1318) without the cytoplasmic domain were generated by PCR subcloning into pcDNA3.1 vector using the above-mentioned forward primer and the following reverse primers; 5′-GATCGGTACCTTACCACTTGTTGTAGTATG-3′ and 5′-GATCGGTACCTTACAGAATGAAAAAGACGC-3′, respectively. All constructs were cloned between *NotI* and *KpnI* restriction sites in pcDNA3.1 vector using the T4 DNA ligase. All constructs were confirmed by restriction digestion and sequencing. Bulk endotoxin-free preparations of all four constructs as well as the empty control plasmid (pcDNA) were prepared for animal studies using a plasmid Giga purification kit (Qiagen).

### *In vitro* Protein expression

Prior to animal experiments, protein expression from all DNA constructs was confirmed *in vitro* in Vero E6 cells (Fig. 1b). Briefly, 80–90% confluent Vero E6 cells in 6-well plates were transiently transfected with 1 μg of each DNA construct (pS, pS∆CD, pS∆TM, pS1, or pcDNA) using FuGENE 6 reagent (Roche) according to manufacturer’s instructions, followed by incubation at 37 °C in a 5% CO_2_ incubator for 48 h. Transfected cells were then washed twice with phosphate-buffered saline (PBS) and lysed with cell lysis buffer as previously described^50^, and subjected to western blot analysis for protein expression using rabbit anti-S1 Abs (Sino Biological). Western blot analysis confirmed that all gene products show bands at expected molecular weights. Notably, the large band that is observed in all blots in Fig. 1b is due to non-specific binding as it was also detected in an un-transfected cell control (data not shown).

### Animal Studies

Six- to 8-week-old female BALB/c mice were obtained from the core facility in King Fahd Medical Research Center (KFMRC), King Abdulaziz University (KAU). All animal experiments were conducted in accordance with institutional guidelines and the approval of the Animal Care and Use Committee at KFMRC. Mice were divided into five experimental groups (5 mice in each group) and immunized on days 0, 14 and 28 with three doses of 100 μg of each construct dissolved in 100 μl PBS. Mice were immunized intramuscularly with two injections (50 μl each) divided between the two thighs. Three weeks after the last doses (day 49), mice were euthanized and blood as well as spleens were collected for immune response analysis.

### ELISA

The end-point titers of anti-S1 total IgG Ab as well as IgG1, IgG2a and IgG2b isotypes from immunized mice were determined by ELISA as described previously^29^,^50^ with minor modifications. Briefly, 96-well plates (EU Immulon 2 HB, Thermo Scientific) were coated with the MERS-CoV S1 protein (Sino Biological) at 2 μg/ml in PBS at 4 °C overnight. Plates were then washed 6 times with PBS containing 0.05% Tween-20 (PBS-T), followed by blocking with 5% skim milk in PBS-T for 1 h at 37 °C. After washing, plates were incubated with a 2-fold serial dilution of mouse sera starting from 1:100 and incubated for 1 h at 37 °C. Then, plates were washed and incubated with peroxidase-conjugated rabbit anti-mouse IgG, IgG1, IgG2a or IgG2b secondary Abs (Jackson Immunoresearch Laboratories) at concentrations recommended by the supplier and incubated for additional 1 h at 37 °C. After extensive washing, Tetramethylbenzidine (TMB) substrate (KPL) was added for 30 min for colorimetric development and the reaction was stopped with 0.16 M sulfuric acid. Absorbance was read spectrophotometrically at 450 nm. End-point titers were determined and expressed as the reciprocals of the final detectable dilution with a cut-off defined as the mean of pre-bleed samples plus three SD.

### Viral microneutralization assay

Microneutralization (MNT) assay was performed as previously described^29^,^30^. Briefly, two-fold serial dilutions of heat-inactivated sera prepared in DMEM starting from a 1:5 dilution were incubated with equal volume of DMEM containing 200 TCID_50_ of MERS-CoV for 1 h at 37 °C in a 5% CO_2_ incubator. The virus-serum mixture was then transferred on confluent Vero E6 cell monolayers in 96-well plates (four wells were used per dilution) and incubated at 37 °C in a 5% CO_2_ incubator. Cytopathic effect (CPE) was observed on days 3 to determine nAb titer. The nAb titer for each sample is reported as the reciprocal of the highest dilution that completely protected cells from CPE in 50% of the wells (MNT_50_).

### CD8^+^ T cell intracellular cytokine staining (ICS)

Memory CD8^+^ T cell IFN-γ responses were evaluated at 3 weeks after last immunizations as previously described^50^. Briefly, single-cell suspensions of splenocytes were prepared from individual mice in each group. Spleens from mice were collected in 10 ml of RPMI 1640 supplemented with 10% FBS and smashed between frosted ends of two glass slides. Processed splenocytes were then filtered through 45-μm nylon filters and centrifuged at 800 g for 10 min. Red blood cells were then lysed by adding 5 ml of ammonium-chloride-potassium (ACK) lysis buffer (Life Technologies) for 5 min at room temperature, and equal volume of PBS was then added. Cells were centrifuged again and pellets were resuspended in RPMI 1640 at a concentration of 1 × 10^7^ cells/ml. Splenocytes were then added to a 96-well plate (1 × 10^6^ per well) and re-stimulated with 5 μg/ml of several synthetic S1 MHC class I–restricted peptides including S291 (KYYSIIPHSI), S319 (QPLTFLLDF), S448 (YPLSMKSDL), S498 (SYINKCSRL), S647 (NYYCLRACV), S703 (TYGPLQTPV), which were synthesized by GenScript as previously described^32^. The stimulation was conducted by incubation for 6 h at 37 °C and 5% CO_2_ in the presence of Protein Transport Inhibitor Cocktail (brefeldin A) (BD Biosciences) according to the manufacturer’s instructions. Stimulated cells were then washed in FACS buffer and stained with LIVE/DEAD Fixable Violet Dead Cell Stain Kit (Invitrogen) and anti-mouse CD8α –FITC antibody (clone 53–6.7; eBiosciences). The cells were then washed with FACS buffer, fixed and permeabilized with Cytofix/Cytoperm Solution (BD Biosciences) according to the manufacturer’s instructions, and labeled with anti–mouse IFN-γ-APC-Cy7 antibody (clone XMG1.2; BD Biosciences). All data were acquired on a BD FACS Calibur flow cytometer and analysis was completed with Flow Jo, Version 8.8.4 (Tree Star Inc.). Results for IFN-γ producing CD8^+^ T cells were calculated as percentage of live CD8^+^ T cells after subtracting the values obtained from no peptide controls from each sample.

### Data analysis

Statistical analysis was conducted using one-way ANOVA. Bonferroni post-test was used to adjust for multiple comparisons between the different groups. All statistical analysis was conducted using GraphPad Prism software (San Diego, CA). P values < 0.05 were considered significant.

## Results

### S1-subunit DNA vaccine induces high levels of anti-S1 Abs in mice

**Figure 2:**
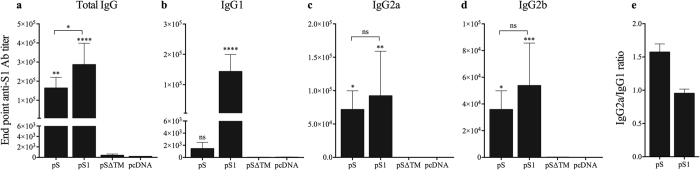
Humoral immune response induced by MERS-CoV Spike DNA vaccines. Circulating MERS-CoV S1-specific Abs were determined at 3 weeks post 2^nd^ boost. End-point titers are shown for (**a**) total IgG, (**b**) IgG1, (**c**) IgG2a and (**d**) IgG2b isotypes. (**e**) IgG1/IgG2a ratio was calculated at 3 weeks after 2^nd^ boosting to determine the type of immune response (Th2 versus Th1) induced by the various constructs. BALB/c mice were i.m. immunized with 100 μg of each construct dissolved in 100 μl PBS on days 0, 14 and 28. A control group was immunized with empty pcDNA vector. Data are shown as mean titer ± s.d. from one experiment out of two independent experiments, with n = 5 mice per treatment group in each experiment. ****P < 0.0001, ***P < 0.001, **P < 0.01 and *P < 0.05 (one-way ANOVA with Bonferroni post-test).

In order to evaluate the immunogenicity of our DNA vaccine candidates, we immunized mice i.m. with three doses of the generated naked DNA constructs (Fig. 1c). To this end, evaluation of Ab levels after one or two doses of naked DNA resulted in no or barely detectable response in all groups consistent with previous report^29^, and thus we only analyzed responses after the last dose. As shown in Fig. 2a, only mice immunized with pS and pS1 but not pS∆TM generated significant levels of systemic S1-specific IgG compared to control group immunized with pcDNA vector. Interestingly, pS1 elicited significantly higher levels of S1-specific total IgG compared to pS immunized mice. It is of note that DNA construct expressing truncated S protein without the cytoplasmic domain (pS∆CD) failed to induce detectable Abs in initial pilot studies; it was not tested further.

### Differential induction of S1-specific IgG isotypes by Spike-based DNA vaccines

We next examined the differences in S1-specific Ab isotypes in the sera of immunized mice in order to determine the quality of the humoral response induced by the different DNA constructs. As shown in Fig. 2b–e, immunization with pS DNA vaccine mainly elicited IgG2a and IgG2b with significantly lower levels of IgG1 isotype, indicating a Th1-biased response (IgG2a/IgG1 ratio of >1.5). As expected, empty vector control (pcDNA) and pS∆TM failed to produce any anti-S1 IgG isotype. On the other hand, plasmid DNA expressing S1 subunit (pS1) induced a balanced Th1/Th2 response (IgG2a/IgG1 ratio of ~1.0) with S1-specific Abs from all isotypes. While IgG2a and IgG2b levels induced by pS1 were significantly higher compared to pS∆TM and empty vector control (pcDNA), no significant difference was observed in the levels of these two isotypes between pS and pS1-vaccinated groups. In contrast, level of S1-specific IgG1 Abs elicited by pS1 vaccine was significantly elevated compared to all groups including pS group. Collectively, compared with the full length S protein, these data suggest that S1 subunit delivered by DNA vector elicited stronger antibody responses and equal ratio of IgG2a/IgG1 whereas the full length S protein induced a Th1-skewed immune response.

### S1-expressing DNA vaccine elicits significant level of IFN-γ response

**Figure 3:**
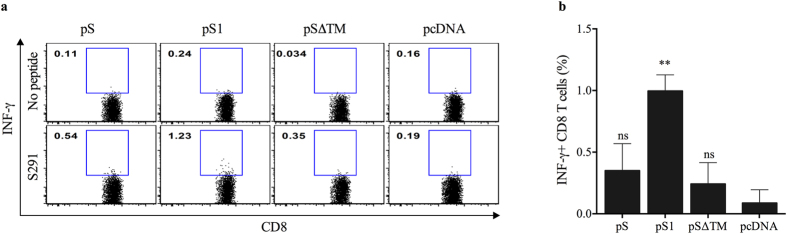
MERS-CoV Spike-specific memory CD8^+^ T cell responses. Immunized BALB/c mice were sacrificed at 3 weeks after 2^nd^ boosting and splenocytes were isolated and re-stimulated *ex vivo* with synthetic S1 peptides for IFN-γ measurement by ICS. Live CD8^+^ T cells were stained for intracellular IFN-γ. (**a**) Flow cytometry plots are representatives from one out of two independent experiments. (**b**) Bar graph represents frequencies of IFN-γ memory CD8^+^ T cells. Data are shown as mean ± s.d from one experiment out of two independent experiments, with n = 3 mice per treatment group in each experiment. **P < 0.01 (one way ANOVA with Bonferroni post-test).

Having observed the Th1-skewed response in pS-immunized mice compared to the pS1 group, we decided to evaluate S1-specific memory CD8^+^ T cell responses by ICS. Remarkably, immunization of mice with pS vaccine did not elicit any significant levels of IFN-γ compared to control group (pcDNA) after re-stimulation with S291 peptide (Fig. 3). On the other hand, re-stimulation of CD8^+^ T cells from pS1-vaccinated animals induced significantly higher levels of IFN-γ compared to all other groups, suggesting that immune-focusing by using S1-based vaccine could not only enhance Ab response but also cell-mediated responses. The inability of pS immunogen to induce S1-specific CD8^+^ T cells IFN-γ was consistent with the overall weaker response compared to pS1-vaccinated group. Interestingly, re-stimulation with several other peptides within the S1 subunit as previously described^32^ failed to elicit any IFN-γ from all groups (Supplementary Figure 1).

### Spike-based DNA immunization elicited cross-neutralizing MERS-CoV Abs against human and camel isolates

**Figure 4:**
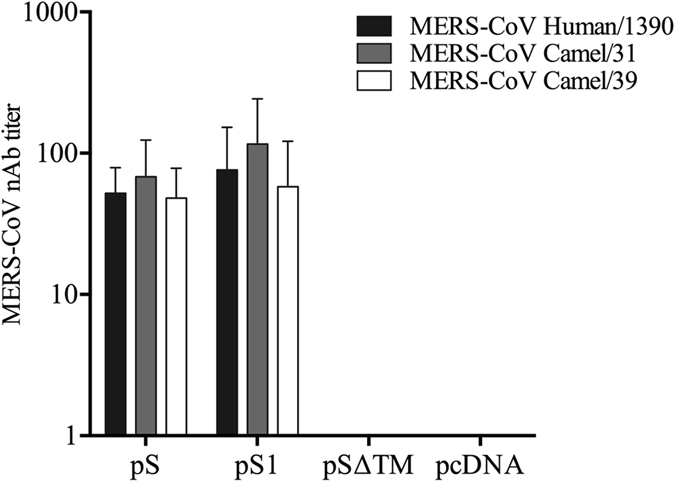
MERS-CoV Spike DNA vaccine induced nAbs. Neutralization titers were determined as the highest serum dilutions from each individual mouse that completely protected Vero E6 cells in at least 50% of the wells (MNT_50_). Titers are shown as means from 5 mice per group ± s.d from one experiment out of two independent experiments.

As our DNA vaccine constructs were made using coding sequence from a 2013 isolate that directly transmitted form infected camel to a human, it was important to test their cross-neutralization activity against recent isolates. To this end, antisera from immunized mice were tested against human and camel MERS-CoV isolates from 2015 and 2016. As shown in Fig. 4, sera collected from mice immunized with DNA expressing full-length S protein or S1 subunit were found to have comparable nAb titers against the human and the camel isolates. These findings clearly show that S protein is a very promising vaccine target as it induced nAbs against human and camel MERS-CoV strains isolated in 2015 (MERS-CoV Human/1390) and 2016 (MERS-CoV Camel/31 and MERS-CoV Camel/39).

## Discussion

The rapid spread and high mortality rate of MERS-CoV infections in several countries of the Arabian Peninsula present a daunting challenge to the international community; the large zoonotic reservoir host of MERS-CoV makes it difficult to eliminate the source of transmission. While public health measures are critical to contain MERS-CoV spread and proven to be effective in limiting outbreaks, development of safe and preventive vaccine is urgently needed.

Several groups have investigated various vaccine platforms to combat MERS-CoV^29^,^30^,^31^,^32^,^33^,^34^,^35^,^36^,^37^,^38^,^39^,^40^,^41^,^42^. Most of these experimental vaccines were based on MERS-CoV full-length or truncated versions of the spike protein; these prototype vaccines were found to have induced high levels of nAbs and sometimes conferred protection against MERS-CoV challenge in several animal models. However, several previous SARS-CoV vaccine studies have also shown that there might be some safety concerns associated with the use of WIV^43^, truncated S subunit/protein vaccines^44^ or vectored vaccines expressing full-length S protein^45^. These concerns included inflammatory and immunopathological effects such as eosinophilic infiltration of the lungs as well as Ab-mediated disease enhancement (ADE) in immunized animals upon viral challenge. It is believed that induction of Th2-polarized immune response and/or non-neutralizing Abs against epitopes within the S protein (i.e. outside the neutralizing-epitope rich RBD or S1 subunit) are the reason for the observed immunopathology and disease enhancement in vaccinated animals^46^,^47^,^48^, suggesting that use of S1 subunit over full-length S protein could be a safer option for vaccine development.

Furthermore, a recent report revealed that MERS-CoV vaccines might be associated with similar type of immunopathologies especially upon induction of Th2-skewed response or use of full-length or truncated S protein^37^. This could be a hurdle facing vaccine candidates expressing non-neutralizing epitopes such as the ones based on full-length S protein^29^. Therefore, immune focusing by using RBD or S1 subunit could represent an attractive approach for safe and effective MERS vaccine. Indeed, several versions of RBD subunit vaccine were tested^38^,^39^,^40^,^41^,^42^ and showed very promising results even upon immunization with very low dose^51^. However, protein subunit vaccines were found to induce skewed Th2 response. Therefore, more studies are needed to develop a safe and approved adjuvant to elicit Th1-skewed response^29^,^47^,^48^.

MERS-CoV DNA vaccines can induce Th1-biased immune response even though multiple injections are usually required due to their low immunogenicity especially in large animals^29^,^30^. Up to date, only two studies have investigated MERS-CoV DNA vaccines by utilizing full-length S protein, which is the primary target of immune response in the host. To dissect the antigenic domains of the S protein, we examined the immunogenicity of naked DNA vaccines expressing several versions of MERS-CoV S protein in mice. We found that pS-immunized group elicited significant IgG2a and IgG2b titers (Th1-skewed response) with very subtle S1-specific CD8^+^ IFN-γ response. On the other hand, pS1-immunization generated markedly increased levels of all IgG isotypes in a balanced Th1/Th2 response along with low but significantly elevated CD8^+^ IFN-γ response compared to pS group. While further animal studies are required to determine whether induction of balanced Th1/Th2 or Th1-biased immune response could aid in the development of safer MERS-CoV vaccine, S1-based vaccines could be a safer option compared to the full-length S-based vaccines.

It is of note that the ELISA plates used for the measurement of binding IgG isotypes were coated with S1 recombinant protein (Fig. 2), therefore, there might be more Abs in the pS vaccinated animals targeting epitopes outside the S1 subunit (i.e. S2 subunit) that were never detected in our analysis. In addition, the observation that both pS1 and pS induced similar nAb titers (Fig. 4) suggests that full-length S protein harbors neutralizing epitopes outside the S1 region as previously reported^29^. The induction of high levels of IgG1 by pS1 vaccine and consequent balanced Th1/Th2 response could probably be explained by the secretion of S1 subunit especially that this immunogen contains the signal peptide without the cell membrane anchoring domains compared to pS vaccine. While additional studies are required to confirm this, we have previously shown that internal viral proteins such as the influenza nucleoprotein could be partially secreted and alter the immune response phenotype when fused to a secretion signal^50^. Furthermore, the weak or undetectable response in pS∆TM and pS∆CD immunized mice is noteworthy especially that Wang *et al*., showed that MERS-CoV DNA vaccine expressing S∆TM induced limited response in mice even after electroporation^29^. Although this response could be due to misfolded protein and rapid degradation of the antigen, or low expression level of these truncated spike proteins given that expression of S∆TM gave low production yields from transfected HEK 293 as previously described^29^, similar vaccines have been shown to be very effective in mice in the case of SARS-CoV^52^.

The finding that immune sera from both pS and pS1 immunized mice could cross-neutralize recent human and camel field isolates is critical. Most previous studies utilized strains such as Jordan-N3 (GenBank ID: KC776174.1) and EMC/2012 (GenBank ID: JX869059.2) in live virus neutralization assay. These viruses were isolated in 2012; they may or may not be same as the currently circulating strains, given that strains used here showed 5–7 and 1–2 amino acid changes in comparison to Jordan-N3 and EMC/2012, respectively (Supplementary Figure 2). Furthermore, several other studies have used pseudovirus neutralization assay to test contemporary strains which may not replicate the actual neutralization breadth against live viruses^29^,^30^. It is of note that the similar levels of nAb titers in both pS and pS1 groups reported here appear to be different from the observation by others, who found significantly higher levels of nAbs induced by a DNA vaccine expressing full-length S protein compared to that expressing S1 protein^29^. However, this discrepancy in results remains to be fully understood but is likely due to the difference in experimental conditions. Specifically, Wang *et al*. utilized electroporation with DNA immunization and pseudovirus neutralization assays to determine nAbs whereas we used naked DNA vaccines and live virus neutralization assays.

Taken together, our study suggests the DNA vaccine expressing S1 subunit could represent a promising candidate vaccine against MERS-CoV while minimizing the risk of the immunopathologies associated with the use of full S protein and Th2 response. However, more studies are clearly required to enhance the immunogenicity of naked DNA vaccine and to examine the safety of this prototype vaccine.

## Additional Information

**How to cite this article:** Al-amri, S. S. *et al*. Immunogenicity of Candidate MERS-CoV DNA Vaccines Based on the Spike Protein. *Sci. Rep.* **7**, 44875; doi: 10.1038/srep44875 (2017).

**Publisher's note:** Springer Nature remains neutral with regard to jurisdictional claims in published maps and institutional affiliations.

## Supplementary Material

Supplementary Figures
