# Supplementary material for: Immunogenicity of Candidate MERS-CoV DNA Vaccines Based on the Spike Protein
Source: Sci Rep. 2017 Mar 23;7:44875. doi: 10.1038/srep44875 (PMC5362948; doi:10.1038/srep44875)
Supplement: Supplementary Figures [file srep44875-s1.pdf]

Immunogenicity of Candidate MERS-CoV DNA Vaccines Based on the Spike Protein

Sawsan S Al-amri[1], Ayman T Abbas[1,2], Loai A Siddiq[1], Abrar Alghamdi[1], Mohammad A. Sanki[3], Muhanna K Al-Muhanna[4], Rowa Y Alhabbab[1,5], Esam I Azhar[1,5], Xuguang Li[6], Anwar M Hashem[1,7]*

[1] Special Infectious Agents Unit, King Fahd Medical Research Center, King Abdulaziz University, Jeddah, Saudi Arabia.

[2]Biotechnology Research Laboratories, Gastroenterology Surgery Center, Mansoura University, Mansoura, Egypt.

[3] Hematology Laboratory, King Abdulaziz University Hospital, Jeddah, Saudi Arabia.

[4] Materials Science Research Institute, National Nanotechnology Center, King Abdulaziz City for Science and Technology (KACST), Riyadh, Saudi Arabia

[5] Department of Medical Laboratory Technology, Faculty of Applied Medical Sciences, King Abdulaziz University, Jeddah, Saudi Arabia.

[6] Center for Vaccine Evaluation; Biologics and Genetic Therapies Directorate; Health Canada; Ottawa, Ontario, Canada

[7] Department of Medical Microbiology and Parasitology Faculty of Medicine, King Abdulaziz University, Jeddah, Saudi Arabia.

*** Corresponding author**

Email: amhashem@kau.edu.sa (AMH)

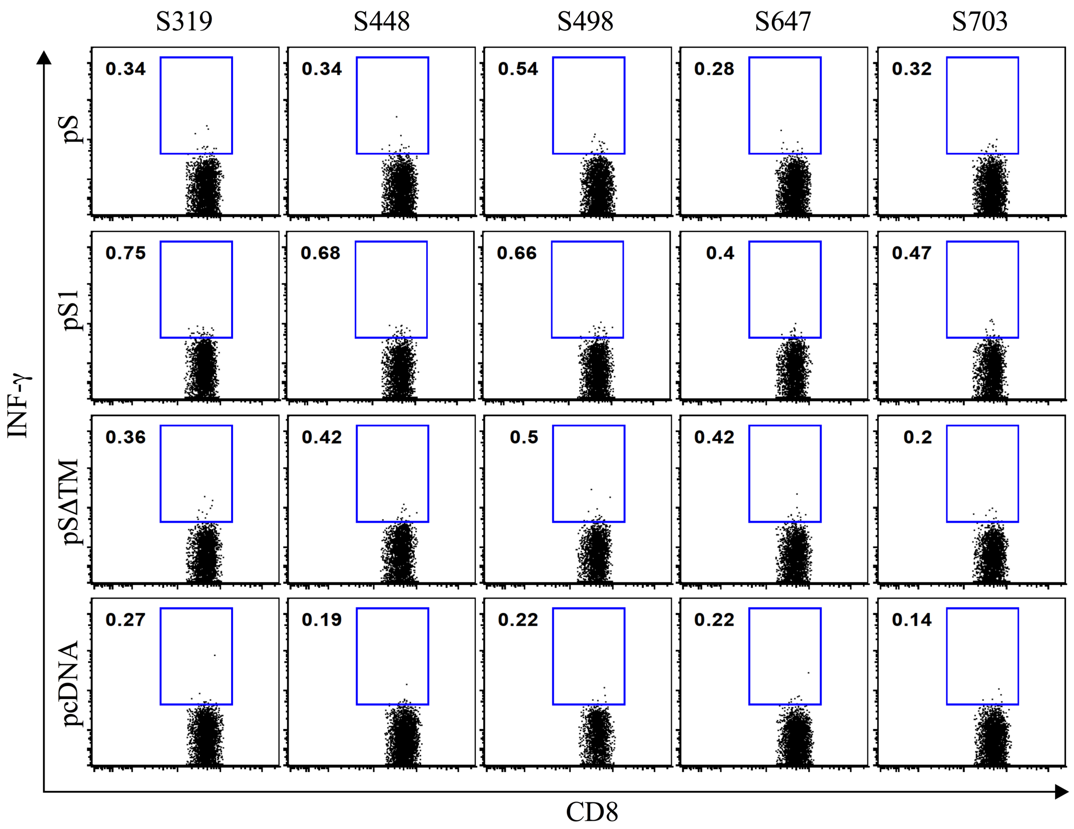

Supplementary Figure 1. MERS-CoV Spike-specific memory CD8$^+$ T cell responses. Immunized BALB/c mice were sacrificed at 3 weeks after 2nd boosting and splenocytes were isolated and re-stimulated *ex vivo* with several synthetic S1 peptides for IFN-γ measurement by ICS. Live CD8$^+$ T cells were stained for intracellular IFN-γ. Plots are representatives from one out of two independent experiments with n = 3 mice per treatment group in each experiment.

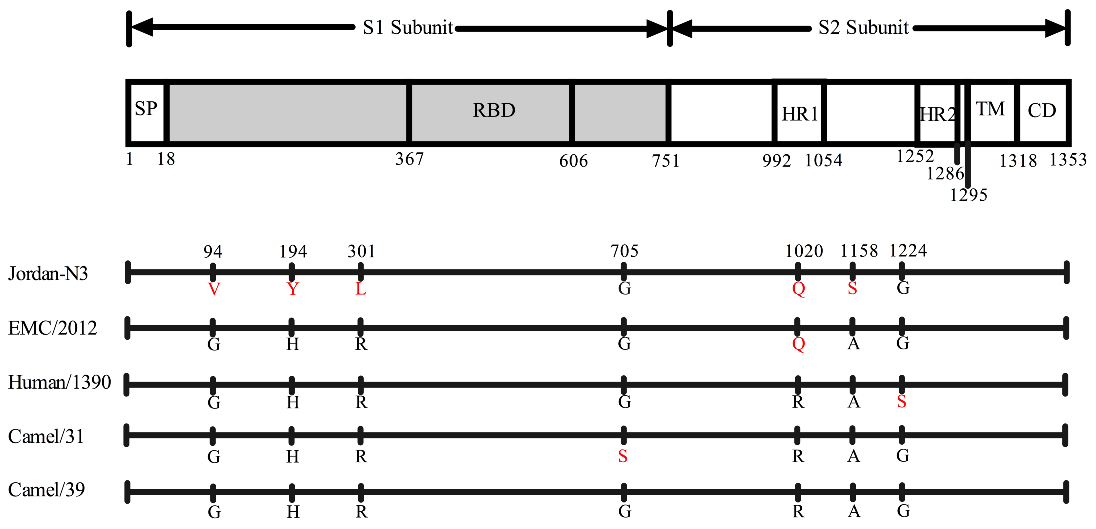

Supplementary Figure 2. Comparison of MERS-CoV Spike glycoprotein sequences across strains used in live virus microneutralization assay. A schematic representation of MERS-CoV S protein is shown with alignment of MERS-CoV S sequences of Jordan-N3 (GenBank ID: KC776174.1) and EMC/2012 (GenBank ID: JX869059.2) as well as the three strains used in this study from 2015 (Human/1390) and 2016 (Camel/31 and Camel/39). The amino acid differences are shown in red in comparison to the consensus sequence. SP: signal peptide; RBD: receptor-binding domain; HR1: heptad repeats 1; HR2: heptad repeats 2; TM: transmembrane domain; CD: cytoplasmic domain.
